# Uncovering the underlying mechanisms and whole-brain dynamics of deep brain stimulation for Parkinson’s disease

**DOI:** 10.1038/s41598-017-10003-y

**Published:** 2017-08-29

**Authors:** Victor M. Saenger, Joshua Kahan, Tom Foltynie, Karl Friston, Tipu Z. Aziz, Alexander L. Green, Tim J. van Hartevelt, Joana Cabral, Angus B. A. Stevner, Henrique M. Fernandes, Laura Mancini, John Thornton, Tarek Yousry, Patricia Limousin, Ludvic Zrinzo, Marwan Hariz, Paulo Marques, Nuno Sousa, Morten L. Kringelbach, Gustavo Deco

**Affiliations:** 1 Center for Brain and Cognition, Computational Neuroscience Group, Department of Information and Communication Technologies, Universitat Pompeu Fabra, Barcelona, 08018 Spain; 2 Sobell Department of Motor Neuroscience & Movement Disorders, UCL Institute of Neurology, London, WC1N 3BG United Kingdom; 3 Wellcome Trust Centre for Neuroimaging, Institute of Neurology, University College London, London, WC1N 3BG United Kingdom; 4 Nuffield Department of Clinical Neurosciences, University of Oxford, Oxford, OX3 9DU United Kingdom; 5 Nuffield Department of Surgical Sciences, University of Oxford, Oxford, OX3 9DU United Kingdom; 6 Department of Psychiatry, University of Oxford, Oxford, OX3 7JX United Kingdom; 7 Center for Music in the Brain, Aarhus University, Aarhus, 8000 Aarhus C Denmark; 8 Lysholm Department of Neuroradiology, National Hospital for Neurology and Neurosurgery, UCLH NHS Foundation Trust, London, WC1N 3BG United Kingdom; 9 Life and Health Sciences Research Institute (ICVS), School of Health Sciences, University of Minho, 4710-057 Braga, Portugal; 10 ICVS/3B’s - PT Government Associate Laboratory, 4710-057 Braga, Portugal; 11 Clinical Academic Center, 4710-057 Braga, Portugal; 12 Instituci Catalana de la Recerca i Estudis Avanats (ICREA), Universitat Pompeu Fabra, Barcelona, 08010 Spain; 13 Department of Neuropsychology, Max Planck Institute for Human Cognitive and Brain Sciences, 04103 Leipzig, Germany; 14 School of Psychological Sciences, Monash University, Clayton VIC, 3800 Melbourne, Australia

## Abstract

Deep brain stimulation (DBS) for Parkinson’s disease is a highly effective treatment in controlling otherwise debilitating symptoms. Yet the underlying brain mechanisms are currently not well understood. Whole-brain computational modeling was used to disclose the effects of DBS during resting-state functional Magnetic Resonance Imaging in ten patients with Parkinson’s disease. Specifically, we explored the local and global impact that DBS has in creating asynchronous, stable or critical oscillatory conditions using a supercritical bifurcation model. We found that DBS shifts global brain dynamics of patients towards a Healthy regime. This effect was more pronounced in very specific brain areas such as the thalamus, globus pallidus and orbitofrontal regions of the right hemisphere (with the left hemisphere not analyzed given artifacts arising from the electrode lead). Global aspects of integration and synchronization were also rebalanced. Empirically, we found higher communicability and coherence brain measures during DBS-ON compared to DBS-OFF. Finally, using our model as a framework, artificial *in silico* DBS was applied to find potential alternative target areas for stimulation and whole-brain rebalancing. These results offer important insights into the underlying large-scale effects of DBS as well as in finding novel stimulation targets, which may offer a route to more efficacious treatments.

## Introduction

Deep Brain Stimulation (DBS) is a remarkably effective treatment for a number of otherwise treatment-resistant disorders including tremor, dystonia, and Parkinson’s disease^1–5^. Initially, target areas for lesional surgery in Parkonson’s Disease were discovered by careful neurosurgical research in humans. Eventually, the highly successful 1-methyl-4-phenyl-1,2,3,6-tetrahydropyridine (MPTP) model in higher primates^6^ helped identify a number of efficacious DBS targets and most importantly the subthalamic nucleus (STN)^7, 8^. Perhaps surprisingly, though, the underlying mechanisms of DBS are not yet resolved despite the fact that DBS in the STN has now been applied to over 150,000 patients. Initially it was thought that, similar to surgical lesions, DBS acted on local circuitry but careful analysis of the biophysical properties of the brain^9^ has shown that the most likely mechanism of DBS is through stimulation-induced modulation of the activity of macroscopic brain networks^10, 11^. Corroborating evidence has come from rodent optogenetic experiments, which have shown that the therapeutic effects within the STN can be accounted for by direct selective stimulation of afferent axons projecting to this region^12^. Still, these studies have not resolved the nature of the whole-brain dynamics arising from DBS.

A principled approach to understanding DBS mechanisms will need to take into account the structural and functional connectivity (FC) of a given DBS target within the diseased brain and to map the ensuing changes caused by this continuous perturbation. Recent advances in computational connectomics have now produced the necessary tools to allow for careful, causal exploration of whole-brain dynamics within the underlying structural connectivity^13–16^. Using these tools, research has demonstrated that there are specific structural “fingerprints” of structural connectivity associated with successful versus unsuccessful outcomes of DBS^17^. In addition, the connectomic analysis of a unique dataset of pre and post-DBS diffusion tensor imaging (DTI) for Parkinson’s disease found significant localized structural changes as a result of long-term DBS^18^. Further, using whole-brain computational modeling on the dataset to track the ensuing changes in functional connectivity of STN, DBS generated Hebbian-like learning in specific STN projections^19^.

Functional connectivity changes following DBS across the whole human brain were first explored using magnetoencephalography (MEG) in patients with DBS for chronic pain which found specific functional changes in brain activity associated with pain relief^20^ as well as the ensuing long-term changes in functional connectivity after 12 months^21^. Subsequent studies have started to use functional Magnetic Resonance Imaging (fMRI), having significantly reduced the risks to the patient^22^ using established safe imaging conditions^23, 24^. A first study demonstrated a reversal in cortico-thalamic coupling during voluntary movements in Parkinson’s disease patients with STN DBS^25^, while a follow-up study used dynamic causal modeling (DCM) of the STN network to further characterize the effective connectivity of resting state motor networks^26^. In another study, fMRI and electroencephalography (EEG) were used to track the changes following DBS of the nucleus accumbens (NAc) in patients with obsessive-compulsive disorder which was found to reduce excessive connectivity between the NAc and prefrontal cortex, with decreased frontal low-frequency oscillations during symptom provocation^27^.

Taken together these studies lend strong support to the idea that therapeutic DBS works by re-balancing the brain activity of the functional and structural networks in the diseased brain^11^. Still, we are missing a mechanistic understanding of how these whole-brain networks change with DBS. In traditional thermodynamical theory, *criticality* refers to a state in which two phases are indistinguishable from one another^28^. From this viewpoint, it has been shown that the resting brain optimally operates in a similar critical manner, at the edge of a bifurcation that represents a transition between states^29^. Here, we used the tools from computational connectomics to investigate the fMRI responses in the right hemisphere of ten Parkinson’s disease patients with DBS ON and OFF compared with 49 Healthy age-matched (as well as 16 non age-matched) participants. This allowed us to explore the local and global impact that DBS has on resting state brain dynamics^30^. The advantage of using this model is that it estimates a bifurcation parameter, which locally (region-by-region) and globally describes whether a system presents asynchronous, critical or synchronous oscillations. Further, to address if turning the stimulation on improves and restores coherence while helping restoring global dynamics back to a Healthy state, we used several metrics that allowed the identification of global enhancements in communicability and synchrony of the network as well producing local oscillatory conditions as a proxy for artificial DBS. In the light of earlier findings addressing large-scale changes caused by Parkinson’s disease^18, 31, 32^ and previous research on DBS mechanisms^33^, we predicted that therapeutic DBS for Parkinson’s disease would create both global and local changes in the large-scale dynamics.

## Results

**Figure 1:**
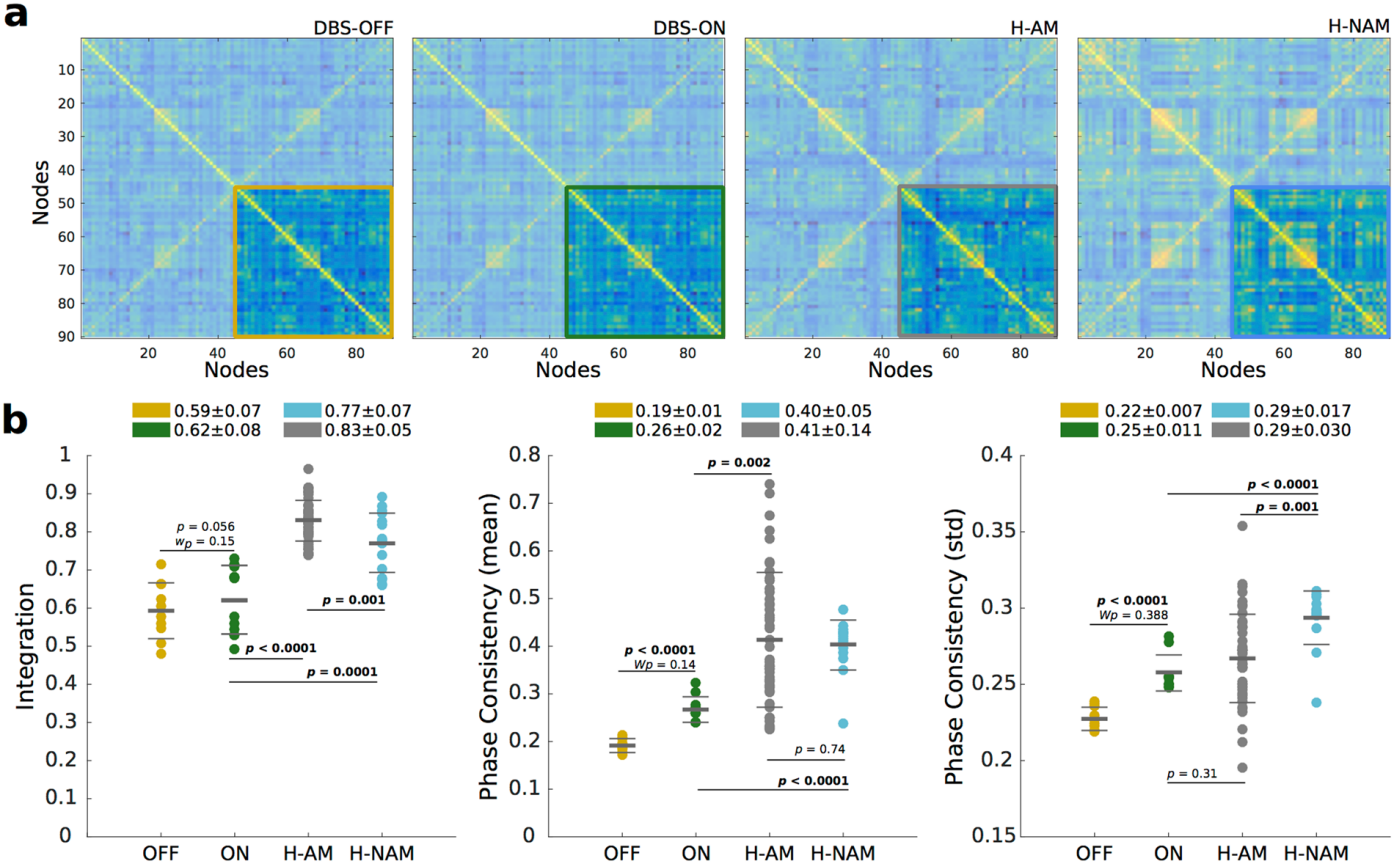
DBS induced changes in global measurements furnishing integration and metastability. These changes were seen in integration, mean phase consistency and mean standard deviation of the phase consistency in both Healthy empirical datasets (H-AM gray, H-NAM light blue) and DBS ON (green) and OFF (orange). (**a**) FC matrices for ON, OFF, H-AM and H-NAM. The right hemisphere in which all analyses were focused is highlighted. (**b**) Metrics for all four groups. Each point represents a participant while mean and standard deviation are described at the top. Differences between ON and OFF correspond to a one-sided paired *t*-test, while differences between ON and Healthy groups correspond to a one-sided unpaired *t*-test. *Wp* represents Levene’s significance.

We first evaluated the differences in whole-brain resting-state FC between Parkinson’s disease patients with DBS OFF and ON and Healthy participants using a number of sensitive methods such as 1) the integration, 2) the mean phase consistency and 3) the standard deviation of phase consistency (see Methods). Overall, these measurements showed low values for DBS OFF, which switched to higher values when DBS was turned ON, approximating the values of the healthy control groups (Fig. 1b). This clear tendency towards healthy values with DBS ON suggests that DBS helps rebalancing resting-state FC on a whole-brain level. Although most of the DBS-ON measures were still lower than the Healthy controls, we found that the standard deviation of phase consistency was restored to a value significantly similar to that of the age-matched healthy controls (H-AM). Note that most significant differences between the healthy control groups are likely to be explained by difference in age, with the H-AM data set showing higher inter-subject dispersion, specifically in phase measurements (Fig. 1b). To control for the possibilities of a tremor confound, we found that motor improvement ratings show no statistically significant correlation with the improvement seen in Parkinson patients after DBS for any of these three metrics (*p* = 0.07, 0.15 & 0.21 respectively; see Supplementary Information).

**Figure 2:**
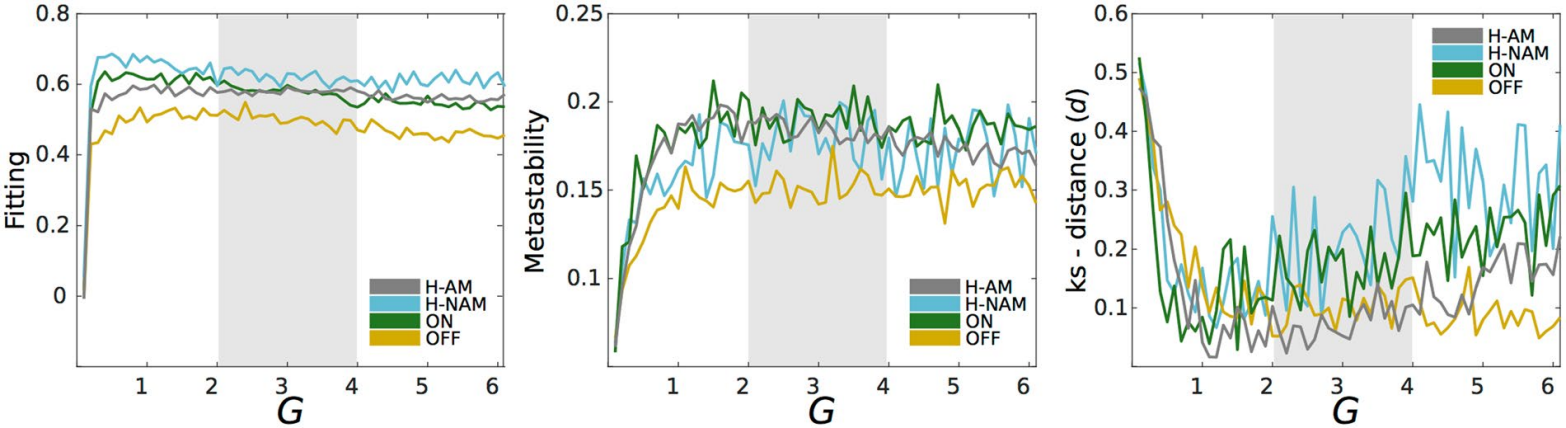
Measuring model fitting. Plots show the agreement metrics between simulated and empirical data for the Healthy (H-AM, gray & H-NAM light blue), ON (green) and OFF (orange) groups. The three panels represent the measurements of fitting, metastability and ks-distance (see Methods) as a function of the coupling strength parameter *G*. The gray area represents the 20 continuous couplings from which the bifurcation parameter values were selected to construct their corresponding distributions.

We then measured the global agreement between the empirical data and the simulated data both in a static and dynamic manner for all groups. As shown in Fig. 2, the fitting between the simulated and empirical FC rapidly increased as a function of the coupling strength *G*, and reached a plateau at around *G* = *2* for both the ON and OFF groups. Here, in accordance with a previous study by van Hartevelt and colleagues (2014) who addressed the positive shift of the global coupling required to simulate a network post-DBS in patients with Parkinson’s disease, the maximum fit is higher in the ON and H-NAM conditions (~0.6) compared to the OFF (0.5) condition and slightly higher in the H-AM group at *G* ~ 2. Considering dynamic aspects, the Kolmogorov-Smirnov distance (ks-d) allows finding the range of *G* where the model better reflects the temporal dynamics of resting-state FC in all groups. We found that the ks-d rapidly decreased with *G* in all groups reaching values of 0.1 also for *G* ~ 2 (Fig. 2), which reflects a better agreement of the dynamic properties of the network. Finally, metastability, which measures fluctuations in the synchrony degree of blood-oxygen-level dependent (BOLD) signals, also showed a similar trend, reaching a plateau after values of *G* ~ 2 for all groups. At this coupling, the ON and both Healthy groups showed a metastability value of around 0.18 while the OFF group presented a value of 0.14, suggesting that DBS restores healthy fluctuations in the synchrony degree, which is corroborated by our findings in the empirical data.

**Figure 3:**
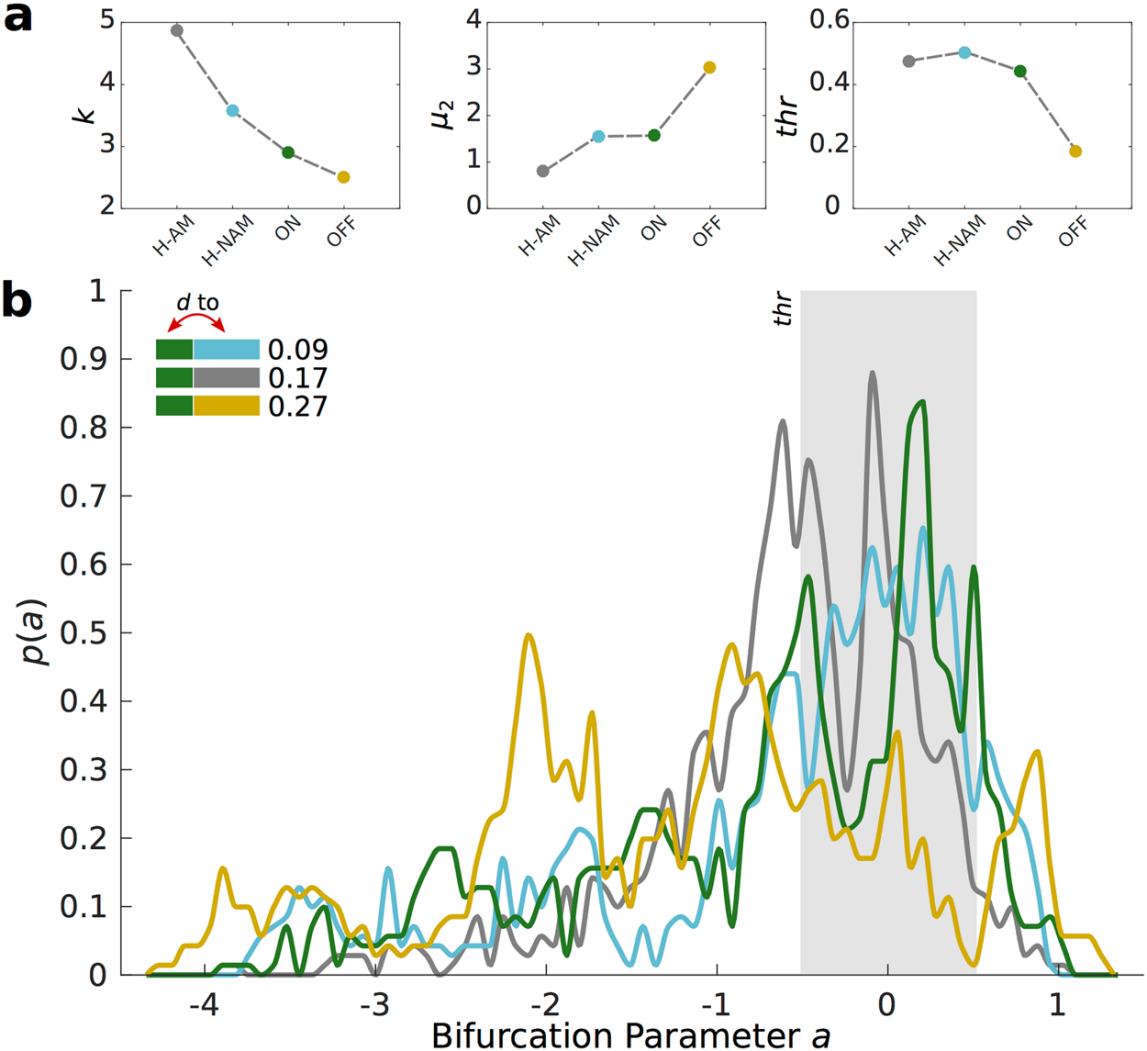
Global bifurcation parameter distributions of ON (green), OFF (orange) and both Healthy (H-AM, gray & H-NAM light blue) groups. (**a**) Global Kurtosis (*k*), second order raw moment (*µ*_2_) and threshold (*thr*) for each of the four distributions. (**b**) Probability density distribution for each condition. The ks-d between ON and the rest of the distributions is described in the top left. The gray area represents a threshold with range −0.5 to 0.5 to count the proportion of bifurcation values centered around 0.

**Figure 4:**
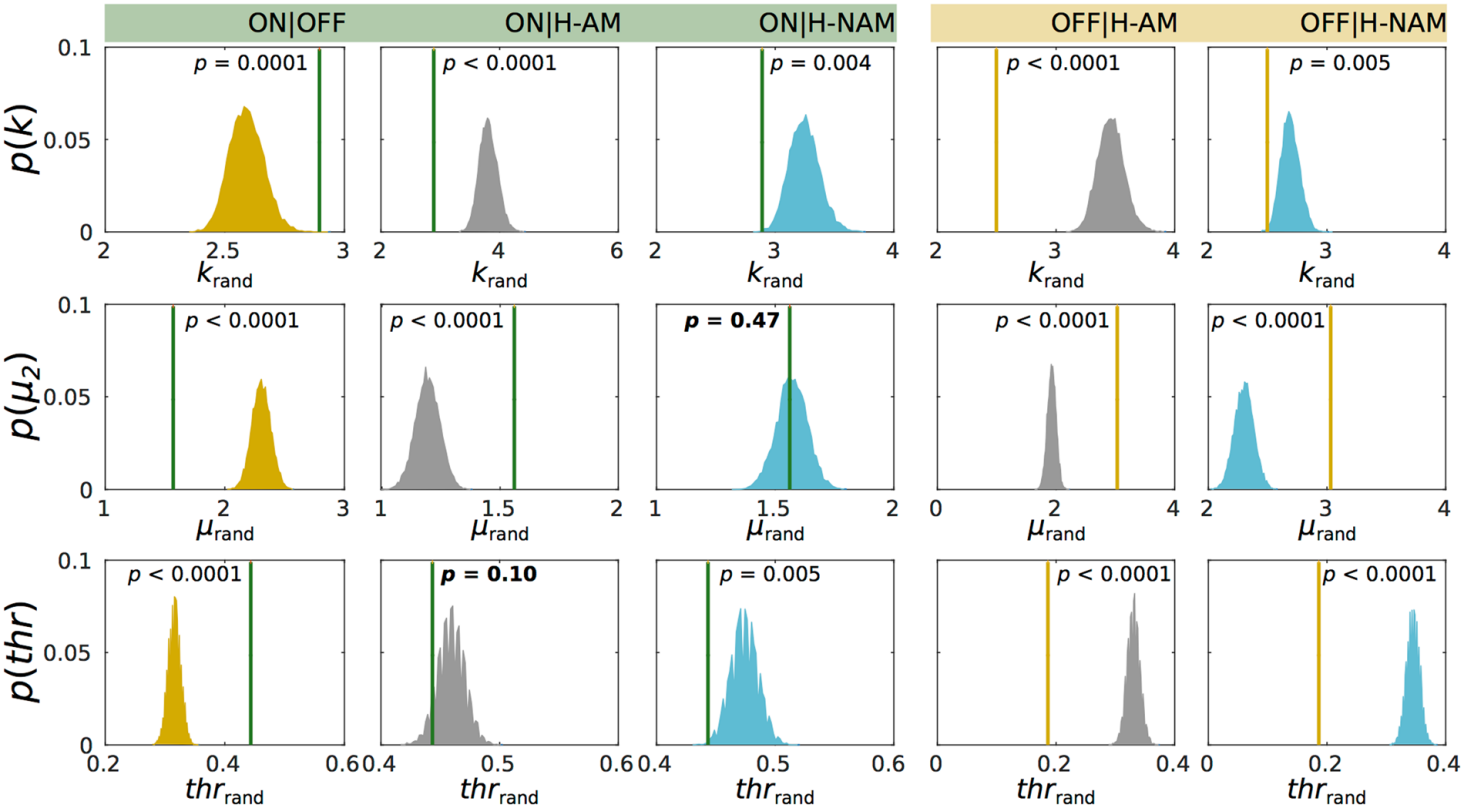
Permutation test for global bifurcation parameter distributions. Five joint distributions are shown: ON|OFF, ON|H-AM, ON|H-NAM, OFF|H-AM and OFF|H-NAM. The observed ON and OFF statistics (*k*, μ_2_ and *thr*) are depicted with a strait line and compared to those of 10,000 randomized surrogate samples (colored distributions) extracted from each of the joint distributions. The proportion of randomized *k*, μ_2_ and *thr* bigger or larger than the observed statistics was used as the significant *p* value.

Next, we evaluated the local dynamics of brain areas by analysing the bifurcation parameters *a*_*j*_ of each area *j*, which were optimized to fit the spectral power of BOLD signals in each participant’s group or condition. Each node *j* has a supercritical bifurcation at *a*_*j*_ = *0*, such that for *a*_*j*_ < *0* the node is in a stable fixed point and is represented by neuronal noise (corresponding to the asynchronous firing of neurons), whereas for *a*_*j*_ > *0* the node switches to a pure oscillatory state (corresponding to the synchronized firing of neurons). The bifurcation parameters of each node optimized for each participant group and condition were extracted from simulations with 20 coupling strength values within the range of optimal *G* defined above (Fig. 2, grey band) (see Methods). Analysing the distribution of bifurcation parameters between groups (Fig. 3), the patients with Parkinson’s disease with DBS-OFF (yellow) displayed mostly negative values of *a*, meaning that the BOLD signals are mostly stable, displaying little fluctuations. In contrast, we found that when DBS was turned ON, the distribution presented sharper peaks skewed towards the bifurcation at *a* = 0 (green), similarly to what we observed in the two Healthy groups (Fig. 3). Analysing in detail the distribution parameters (Fig. 3a) this is reflected by higher *k*, lower μ_2_ and higher *thr* values in the ON and Healthy conditions compared with OFF. When nodes are operating close to the bifurcation (*a~0)*, noisy fluctuations may induce temporary excursions to the oscillatory regime leading to the emergence of oscillations, hence increasing the corresponding spectral power. Investigating the *ks* distances (*d*) between parameter distributions we found smaller distance between Healthy and DBS ON distributions (Fig. 3b). The permutation test showed significant differences between most of the metrics, (Fig. 4). Interestingly, the only two comparisons that did not pass the significance test were ON µ_2_ compared with µ_rand_ from pooled ON|H-NAM (*p* = 0.47) and ON *thr* compared with *thr*_rand_ from pooled ON|H-AM (*p* = 0.10), suggesting a rebalance towards the healthy regime when DBS was turned ON.

**Figure 5:**
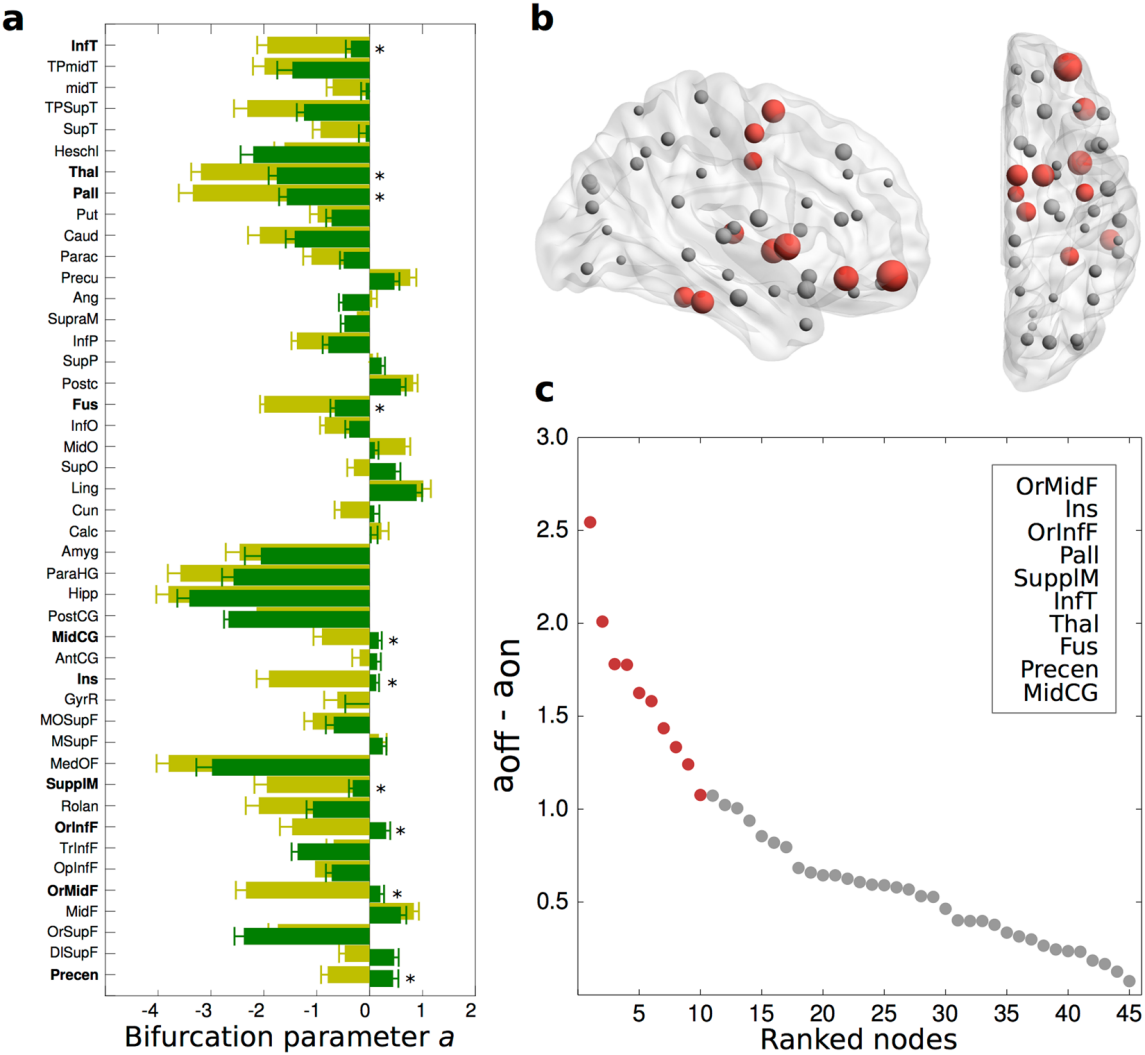
Local differences in bifurcation parameter values for Parkinson’s disease patients. (**a**) Here we show the full bar plot of the mean ± standard deviation bifurcation parameter value in each of the 45 nodes from the right hemisphere for both the ON (green) and OFF (orange) condition. Stars and bolded regions highlight nodes with the most pronounced shift. (**b**) Sagittal and axial view of a brain depicting the absolute bifurcation parameter shift of all nodes. Size represents shift magnitude and red nodes are those ranking in the top 10 with the largest shift. (**c**) Bifurcation parameter shift represented as the absolute difference between *a*_off_ and *a*_on_. Top 10 nodes are depicted in red and listed in the top-right insert. Table 2 shows the full names of abbreviated brain regions within the AAL parcellation shown. 3D brain generated with BrainNet Viewer^79^.

We also inspected the bifurcation parameters across nodes, which allowed the identification of significant local changes between the ON and OFF conditions (Fig. 5a). Regions such as the thalamus and the globus pallidus presented large shifts from being highly asynchronous (*a*_*j*_ << 0) in the OFF condition to values nearer criticality (*a*_*j*_~0) with DBS ON, and ranked within the top 10 nodes with the most pronounced bifurcation parameter change (Fig. 5b,c). Interestingly, these regions are two of the main targets of DBS for Parkinson’s disease treatment^34–36^. Other regions also ranking within the top 10 nodes with the most pronounced shift are the supplementary motor area, middle cingulate gyrus as well as the insula, and the orbital part of the middle and inferior frontal gyrus switching from asynchronous in the OFF condition to values of almost 0 and even oscillatory in the ON condition (Fig. 5a,c). In contrast, only the posterior cingulate, the orbital part of the superior frontal gyrus, the triangular part of the inferior frontal gyrus and Heschl’s gyrus presented the opposite switching, from less asynchronous in the OFF to more negative in the ON condition (Fig. 5a). On the other hand, many regions such as the olfactory cortex, amygdala and hippocampus showed a higher oscillatory behaviour in the OFF condition and slightly decreased towards a bifurcation parameter value of 0 in the ON condition suggesting that DBS is pushing brain dynamics closer to a critical regime, which we found to be a characteristic of resting-state activity in healthy participants.

**Figure 6:**
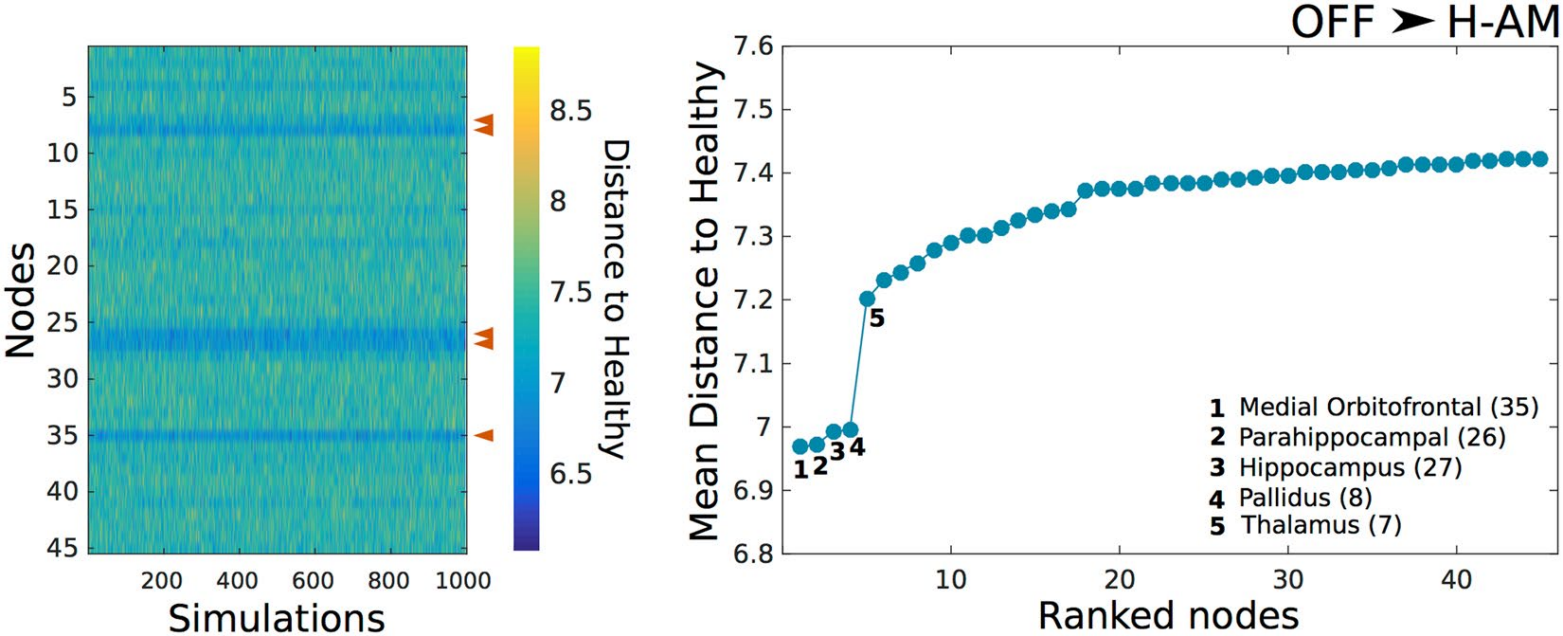
Euclidean distance to Healthy from OFF after artificial DBS. Color map depicted in the left represents the distance to H-AM from OFF after artificial DBS in each of the 45 nodes across all simulations. The top 5 regions with the lowest mean distance are indicated with a red arrow. The mean Euclidean distance ranked from lowest to highest and the top 5 nodes are depicted in the right plot. Numbers in parentheses indicate the region in Table 2.

Finally, our artificial *in silico* representation of local DBS showed that some regions contribute more in shifting global dynamics of DBS OFF towards a Healthy regime as depicted by the mean Euclidean distance between the evoked (see Methods) and the Healthy reference bifurcation vectors (Fig. S2). Remarkably, some of these regions were the thalamus and globus pallidus for H-AM (Fig. 6). As for the non age-matched group, the top regions shifting OFF dynamics to Healthy were the putamen, caudate nucleus and the supplementary motor area (Fig. S3). All of the above mentioned areas are clearly involved in Parkinson’s disease therapy^36–41^.

## Discussion

The research presented here has led to novel insights into the mechanisms of DBS, using computational connectomics to model the large-scale changes elicited by therapeutic DBS in Parkinson’s disease. We studied neuroimaging data from ten Parkinson’s disease patients and found that the overall working point of the brain is shifted towards a critical, Healthy state by turning DBS ON in patients with Parkinson’s disease. Importantly this model was used as a first step to find ways of applying DBS to the diseased brain *in silico* and carefully explore alternative stimulation sites to move the system back to the Healthy regime. Overall, the modeling of direct empirical observations using fMRI significantly improves our understanding of the underlying DBS mechanisms.

Only a handful of studies have analyzed the impact that DBS has on global brain activity in patients suffering from Parkinson’s disease. Kahan *et al*.^26^ found that the overall effective connectivity of motor cortico-striatal and thalamo-cortical pathways is increased by DBS. Interestingly, this enhancement of connectivity strength was accompanied by reduction of clinical impairment. Owing that functional connectivity patterns are disrupted in the default mode network in patients with Parkinson’s disease^32, 42^ and that resting state effective connectivity seems to be reshaped by DBS^25, 26^, the results of the present study point towards large-scale rebalancing by DBS^18, 43^. Among other things, this provides a better mechanistic understanding of the putative long-lasting functional impact of deep brain stimulation, previously demonstrated in a unique study of a Parkinson’s disease patient with measurements of structural DTI presurgical brain changes compared to after six months of treatment^18, 19^, which also showed that the working point of the brain can be partially restored by DBS. Notably, within the context of the model used, DBS seems to push the system towards a regime that is no only more similar to a Healthy state, but also centered around the bifurcation (Figs 3 and 4), which again indicates that proper brain functioning might by linked with criticality^29^. Results showing that phase consistency is higher for DBS ON than DBS OFF (Fig. 1), suggest that activity in Parkinson’s disease is more rigid and less variable while DBS helps creating a more flexible state. This notion is supported by a study that found that whole-brain activity in Parkinson’s disease is characterized by a more random and less efficient state^44^.

Despite the fact that the exact mechanisms of how local bifurcation dynamics affect global characteristics remain hidden, these global changes were mirrored by specific local changes. We found, for example, that DBS led to shifting the bifurcation parameters of the thalamus and the globus pallidus from negative to values nearer the bifurcation, which means that their oscillatory power is increased (Fig. 5). It is well known that the severe degradation of the dopaminergic system causes hyperactivity in the globus pallidus, which strongly affects motor function^45^. These regions are also common targets of DBS for Parkinson’s disease and essential tremor^36^ and have been shown to have significant therapeutic impact on alleviating motor symptoms^35, 46^ while unilateral pallidotomy studies have also shown alleviation of motor symptoms and metabolic increase measured by Position Emission Tomography in the primary motor, lateral premotor and dorsolateral prefrontal cortex^47, 48^. Following the same tendency, the supplementary motor area changed from asynchronous to a near critical behavior, while the precentral gyrus completely switched from asynchronous to stable and both ranked within the top 10 nodes displaying the largest bifurcation parameter shift (Fig. 5c). This is potentially of interest, given that a study found that Transcranial Magnetic Stimulation (TMS) of the supplementary motor area helps alleviating motor symptoms in patients with Parkinson’s disease^40^. Although a different region, in line with these findings a recent study showed that DBS successfully reduces excessive neuronal phase-locking interactions during resting state throughout the motor cortex^49^. Further, the insula also presented an evident shift from asynchronous to critical behavior, which is noteworthy given its tight link with non-motor symptoms in Parkinson’s disease^50^, as well as the previously reported BOLD signal increases seen in the insula during voluntary movements under STN DBS^25^.

Previous studies have confirmed local synchronized oscillatory behavior in the beta frequency in the subthalamic nucleus of patients with Parkinson’s disease that is ameliorated by therapeutic replacement of L-dopa or in the presence of therapeutic STN DBS^51, 52^. Interestingly, our results suggest that at the local level DBS is also pushing some brain areas towards a less oscillatory state as seen in, for example, the posterior cingulate, Heschl’s and the orbital part of the superior frontal gyrus where the bifurcation parameters switched from less to more asynchronous states. Again, this functional result fits well with structural findings, e.g. by van Hartevelt and colleagues (2014) who found that all three regions present higher nodal efficiency post-DBS. The fact that many other regions such as the precuneus, angular and middle frontal gyrus presented slightly more positive values in the OFF compared to the ON condition, but that overall metrics were more centered around the bifurcation (a_j_~0) again indicates that, whether it is changing the local area dynamics from a stable fixed point to the oscillatory regime or vice-versa, DBS is enhancing communicability and efficiency by pushing the system towards criticality.

Recording methods that can capture activity at faster time scales compared to fMRI such as electrocorticography (EcoG) and EEG have shown that Parkinson’s disease is represented by hypersychronization on the beta band (8–35 Hz) in the sensorimotor network and the STN^53^, which is interestingly restored both after DBS^54, 55^ and by dopaminergic therapy^56, 57^. Still, it is well recognized that DBS at the STN is able to improve motor symptoms that persists in medicated patients and that the effect is long-lasting^58, 59^ which represents a significant improvement in quality of life compared to medicated patients^60^. Additionally, local field potential (LFP) recordings have shown strong synchronization in the basal ganglia in patients with Parkinson’s disease^61–63^. Although informative on temporal aspects, all these studies only offer local mechanistic interpretations. At a whole-brain scale, a study that used MEG did find that network organization in Parkinson’s disease is shifted towards a random structure representing a less efficient state^44^. In addition, the same study found that through multiple frequency bands, node efficiency is reduced in orbitofrontal parts^44^ while another recent study used MEG to show that DBS increases gamma power in frontal cortices, which negatively correlated with motor symptom alleviation^64^. This is interesting and in line with our findings as although locally Parkinson’s disease seems to be represented by hypersynchrony^61–63^, globally and on a large scale, brain dynamics in Parkinson’s disease are less predictable and less efficient.

As a first proof of concept in finding novel and more efficacious targets for DBS in Parkinson’s disease without clinical intervention and directly informed by computational models, we showed that forcing local stable oscillatory conditions in some regions pushed the system closer to the Healthy regime (Fig. 6). Remarkably and unexpectedly, two of the top regions shifting the system closer to the Healthy age-matched group were the thalamus and globus pallidus (Fig. 6), both common targets of DBS in Parkinson’s disease^34–36^. Other areas ranking high were the orbitofrontal, hippocampal and parahippocampal gyrus, rising interesting questions about the clinical utility of stimulation in these regions as it might point to the need for relieving two of the cardinal symptoms of PD, namely early olfactory deficits and dementia^65^. As for the H-NAM group, equally interesting results were observed as both the putamen and caudate nucleus ranked high (Fig. S3), which are both part of the basal ganglia and clearly involved in Parkinson’s disease^38, 39, 66^. Accordingly, a study found that DBS in the putamen might help improve motor symptoms in Parkinson’s disease^38^, although the accuracy of the stimulation location has been questioned^67^. The application of *in silico* DBS aims has great potential for using computational models to find new target stimulation areas. While we focused on group-level differences, moving towards a possible clinical application requires applying this method in single participants.

It should also be noted that future work should try to control confounds potentially introduced by the resting hand tremor in patients when DBS is turned off. This is evident, for example, in patients 9 and 10 (Table 1), as their change in motor improvement ratings while DBS ON compared with OFF were less noticeable in contrast with other patients. Despite this, clinical improvement ratings did not correlate with any of the three large-scale metrics used (see Supplementary Information), reducing the probabilities of a tremor confound. Furthermore, it will be important to replicate our modeling findings using control participants scanned on the same scanner, despite the fact that both Healthy and ON parameter profiles are all centered around the bifurcation (Figure 3) adding important validity to the modeling procedure used here.

**Table 1:** Detailed Patient information.

Patient	Age	Sex	Dominant hand	Months since surgery	UPDRS-III		Right electrode				Left electrode	
					Off/OFF	Off/ON	Volts	Pulse width	Freq/Hz	Volts	Pulse	Freq/Hz
								/μs			Width	
											/μs	
1	65	F	R	20	53	21	0.5	60	180	3.3	90	180
2	54	F	R	9	33	10	2.4	60	130	2.4	60	130
3	65	M	R	67	60	20	3.7	60	130	3.45	90	130
4	50	F	L	102	51	17	3.8	60	185	3.6	60	185
5	54	M	R	19	45	26	2.4	60	130	2.3	60	130
6	56	M	L	30	52	19	3.6	90	145	3.3	90	145
7	43	M	L	48	51	23	5.4	60	80	4.1	60	80
8	61	M	R	8	46	25	3.2	60	130	2.9	60	130
9	56	M	R	28	44	42	3.7	60	130	4.1	60	130
10	45	M	R	48	53	44	2.4	60	130	3.15	60	130
**Mean**	**54.9**			**37.9**	**48.8**	**24.7**	**3.1**	**63**	**137**	**3.26**	**69**	**137**
**SD**	**7.5**			**29.3**	**7.3**	**10.6**	**1.3**	**9.5**	**29.4**	**0.6**	**14.5**	**29.4**

Finally, the lack of reversibility from DBS ON to OFF also represents an important limitation. Importantly, a study using an off-on-off protocol showed that therapeutic DBS quickly reduced phase-amplitude interactions (2–4 seconds after stimulation) while this reduction disappeared after minutes of turning the stimulation off^49^, suggesting that neural dynamics elicited by DBS quickly appear, but present a degree of robustness and resilience once present. The observation of quick phase-amplitude reduction is in line with our findings showing fast rebalancing of brain dynamics from the DBS OFF to the Healthy regime.

In this study, we have explored the impact that therapeutic deep brain stimulation has on large-scale brain dynamics. Remarkably, we were able to show that DBS shifts the overall brain dynamics towards the bifurcation (rather than towards noisy or asynchronous oscillatory states) and thus closer to the dynamical regime found in the Healthy brain. This is further supported by our findings of an enhancement in global synchrony and integration by DBS. Importantly, we finally showed that forcing local stable oscillatory conditions in some regions as a proxy for DBS pushes the system closer to the Healthy regime, especially at regions such as the thalamus, globus pallidus and caudate nucleus. Future studies are required to further clarify the mechanisms underlying these local changes leading to global enhancement and whether there may in fact be other DBS targets that can better rebalance the brain dynamics back to a Healthy state.

## Methods

### Ethics

Scanning of all participants in all three groups was performed in accordance with the Declaration of Helsinki (59th amendment). The scanning of patients was approved by the National Hospital and Institute of Neurology Joint Ethics committee (approval number 09/H0716/51). All patients provided written informed consent. The scanning of the age-matched Healthy group was approved by national and local ethics review boards (Comissão Nacional de Protecção de Dados, Hospital de Braga, Centro Hospitalar do Alto Ave and Unidade Local de Saúde do Alto Minho). All volunteers signed informed consent and all medical and research professionals who had access to participants’ identity signed a Statement of Responsibility and Confidentiality. Scanning of participants on the second Healthy control group was approved by the internal research board at CFIN, Aarhus University, Denmark. Ethics approval was granted by the Research Ethics Committee of the Central Denmark Region (De Videnskabsetiske Komitéer for Region Midtjylland). All healthy participants provided written informed consent.

### Data acquisition

#### Patients

We studied ten patients (Table 1) who met the UK brain bank criteria for idiopathic Parkinson’s disease, and had received bilateral STN DBS for more than 6 months. All surgeries were performed at the National Hospital for Neurology & Neurosurgery (NHNN), Queen Square, London, using stereotactic T2-weighted MRI for both preoperative targeting and immediate postoperative verification of electrode contact locations confirming that they were well-sited within the STN (using Model 3389, Medtronic) prior to implantation of the extension cables and the implanted pulse generator (IPG, Kinetra^TM^, Medtronic)^68, 69^. Stimulation parameters were set to produce optimal clinical responses. Medication was withdrawn overnight (10–12 hours) before scanning. We limited inclusion to those patients who could tolerate lying flat with minimal head tremor while being both OFF medication and for DBS OFF.

For each patient, before scanning, both ON and OFF stimulation, we recorded the Unified Parkinson’s disease Rating Scale part III (UPDRS-III) scores (clinical measure of Parkinson’s disease motor impairment; higher score confers greater impairment). A detailed score breakdown can be found in Supplementary Table 1. In addition, we also noted system impedance and stimulation parameters. Importantly, we reset the IPG counters before scanning as the IPG monitors how many times it has been switched ON and OFF. This allowed us to check the counter after scanning to ensure that the pacemaker was not accidentally turned ON or OFF during scanning.

#### Healthy participants

Two sets of healthy participants were used in this study. The first group (H-AM) was recruited in the University of Minho, Portugal. Importantly, this group was large (49, 30 males) and age-matched (57.95 + /− 4.05) with Parkinson Patients. We also included participants from a Healthy non-aged matched group (H-NAM) who were recruited through the online recruitment system at Aarhus University. Neuroimaging data were collected at CFIN, Aarhus University, Denmark, from 16 Healthy right-handed participants (11 men and 5 women, mean age: 24.75+/−2.54). Participants with psychiatric or neurological disorders (or a history thereof) were excluded from participation in the study.

#### Magnetic resonance imaging data acquisition for patients

Overall, the scanning of patients was performed at NHNN, using the safe protocol previously published^22–24^. Specifically, the scanning of patients was performed in a Siemens Avanto 1.5T MRI scanner using a transmit-receive (Tx/Rx) head coil following on-site tissue-equivalent test-object thermometry experiments confirming that, under strict protocol, sequences used in functional MRI studies posed no risk to the patient^23^. As such the specific absorption ratio in the head was limited to less than 0.1 W/kg.

Patients were scanned both during active therapeutic (ON) and inactivated stimulation (OFF). The order of data collection (i.e. ON stimulation then OFF stimulation, and vice versa) was randomly assigned, such that half the patients were scanned ON then OFF, and half were scanned OFF then ON. All patients had their stimulation switched off approximately an hour before scanning for a brief trial lasting approximately 10 minutes before entering the scanner. Their stimulation was then switched back on to allow for safe transfer into the scanner. Stimulation was then switched off approximately 15 minutes before the OFF condition resting state data collection took place.

The scanning order was counterbalanced across patients who received three functional MRI scans during each stimulation condition: (i) resting state with eyes closed (Whole-brain echo planar imaging (EPI): repetition time (TR) = 2420 ms; echo time (TE) = 40 ms; flip angle = 90°; field of view = 192 × 192 mm^2^; matrix size = 64 × 64; 32 axial slices 3.5 mm thick, gap between slices of 0.7 mm; spatial resolution = 3 × 3 × 4.2 mm^3^; duration = 8 min; 200 scans); (ii) motor task (right hand); and (iii) motor task (left hand). To securely support the head and limit head movement, a vacuum moulded cushion was used and patients were able to activate an alarm if they experienced discomfort. As is common, the connection between the electrode lead and the extension cable, which are often placed above the left parietal bone caused a loss-of-signal artifact which resulted in data not being acquired in left hemispheric sensorimotor areas^18, 25, 26^.

In this study, we used only the data from the two resting state sessions (DBS ON and OFF), in addition to two field map scans and an anatomical T1-weighted MP-RAGE structural scan for each patient. After scanning, active stimulation was restored, normal medication was administered, and a UPDRS-III examination was repeated to confirm patients had returned to their clinical baseline. As described above, the settings, counters and impedance of the DBS system were recorded to confirm there were no additional activations induced by the scanner.

#### Magnetic resonance imaging data acquisition for Healthy participants

Age-matched group (H-AM): Prior to the acquisition, participants were instructed to remain still, with eyes closed, not to fall asleep and not to think of anything in particular. The resting fMRI acquisition was performed using a clinical approved 1.5T Siemens Magnetom Avanto (Siemens Medical Solutions, Erlangen, Germany) MRI scanner with a 12-channel receive-only head coil at Hospital de Braga (Portugal). A BOLD sensitive echo-planar imaging (EPI) sequence was used with the following parameterization: 30 axial slices, TR/TE = 2000/30 ms, FA = 90°, slice thickness = 3.5 mm, slice gap = 0.48 mm, voxel size = 3.5 × 3.5 mm^2^, FoV = 1344 mm and 180 volumes.

*Non age-matched group* (*H-NAM*): Participants were scanned in one session on a 3 T Siemens Skyra scanner at CFIN, Aarhus University, Denmark. The parameters for the structural MRI T1 scan were as follows: voxel size of 1 mm^3^; reconstructed matrix size 256 × 256; TE of 3.8 ms and TR of 2300 ms. The resting-state fMRI data were collected using whole-brain EPI with TR = 3030 ms, TE = 27 ms, flip angle = 90°, reconstructed matrix size = 96 × 96, voxel size 2 × 2 mm with slice thickness of 2.6 mm and a bandwidth of 1795 Hz/Px. Approximately seven minutes of resting state data were collected per participant.

Resting fMRI standard preprocessing was performed with FMRIB Software Library tools (FSL v5.07; http://fsl.fmrib.ox.ac.uk/fsl/). A detailed processing description for creating the FC matrices can be found in the Supplementary Information. In short, resting state BOLD signal fluctuations were used to compute matrices of subject-specific FC between 90 non-cerebellar brain areas, defined according to the AAL template^70^ from which only the right hemisphere (Table 2, Fig. S1) was used for the analysis to avoid artifacts arising from the connection between the electrode lead and the extension cable in the left hemisphere of the patients.

**Table 2:** AAL regions and abbreviations.

AAL Region	Abbreviation
Inferior temporal gyrus	InfT
Temporal Pole: middle temporal gyrus	TPmidT
Middle temporal gyrus	midT
Temporal Pole: superior temporal gyrus	TPSupT
Superior temporal gyrus	SupT
Heschl’s gyrus	Heschl
Thalamus	Thal
Globus pallidus	Pall
Putamen	Put
Caudate nucleus	Caud
Paracentral lobule	Parac
Precuneus	Precu
Angular gyrus	Ang
Supramarginal gyrus	SupraM
Inferior parietal gyrus	InfP
Superior parietal gyrus	SupP
Postcentral gyrus	Postc
Fusiform gyrus	Fus
Inferior occipital gyrus	InfO
Middle occipital gyrus	MidO
Superior occipital gyrus	SupO
Lingual gyrus	Ling
Cuneus	Cun
Calcarine Fissure	Calc
Amygdala	Amyg
Parahippocampal gyrus	ParaHG
Hippocampus	Hipp
Posterior cingulate gyrus	PostCG
Middle cingulate gyrus	MidCG
Anterior cingulate gyrus	AntCG
Insula	Ins
Gyrus rectus	GyrR
Superior frontal gyrus, medial orbital	MOSupF
Superior frontal gyrus, medial	MSupF
Medial OFC/Olfactory	medOF
Supplementary motor area	SupplM
Rolandic operculum	Rolan
Inferior frontal gyrus, orbital	OrInfF
Inferior frontal gyrus, triangular	TrInfF
Inferior frontal gyrus, opercular	OpInfF
Middle frontal gyrus, orbital	OrMidF
Middle frontal gyrus	MidF
Superior frontal gyrus, orbital	OrSupF
Superior frontal gyrus, dorsolateral	DlSupF
Precental gyrus	Precen

#### Phase consistency

To assess dynamical properties in functional connectivity, we first filtered the time series with a band-pass of 0.04–0.07 Hz (which contains more functionally relevant information than other bands^71–74^). Next, by using a Hilbert transform, we created a phase coherence matrix by evaluating the instantaneous phase at each time point *t* of every node *j* and then computing the phase difference across all nodes. Finally, by using a three-step sliding window technique, we measured the similarity of the phase coherence matrices over *t* to reconstruct a dynamical FC or *phase consistency matrix*, which was then summarized in terms of its mean and standard deviation. The latter characterizes fluctuations in phase synchronization and furnishes a measure of metastability. We reconstructed this phase consistency matrix in each of the 10 participants for both the ON and OFF condition as well as in each of the Healthy participants in both groups. Metrics between the ON and OFF condition were compared with a one-sided paired *t*-test, while between the ON and Healthy with a two-tailed unpaired *t*-test.

#### Integration

The integrative features of the network were measured using two different metrics. The first one is a simple but rather powerful way to address how integrated or coupled a network is. First, a connectivity threshold *c* (goes from 0 to 1 in steps of 0.01) is gradually applied for any given FC until the matrix is fully disconnected. Then, the size of the largest component *L* in which all pair of nodes are connected is calculated for every thresholded matrix. Finally, we defined integration *I* as:

1 $$I=(0.01)\frac{1}{N}\sum _{c}^{1}{L}_{c}$$

where *N* is the number of nodes in the network and *L*_*c*_ is the thresholded largest component. This was applied for all participants in both the ON and OFF conditions and compared the values with a paired *t*-test. We further calculated this same metric in both Healthy groups as a way to quantify a baseline control integration value and compared it to the ON condition with an unpaired *t*-test.

In addition to compute a paired *t*-test to compare the ON and OFF distributions of the three metrics described (integration, mean phase consistency and phase consistency dispersion), we used a Lavene’s test to make sure that the standard deviation in both groups was not significantly different from one another.

#### Whole-brain Modeling

The dynamic whole-brain model uses a DTI-based structural backbone network^18^ of 45 brain regions (Table 2). A detailed methodological description can be found in two recently published studies^71, 72^ and in the Supplementary Information. In short, this model has two sorts of parameters: a bifurcation parameter that is local to each node and a free parameter, which scales the global connectivity (coupling). Heuristically, we can consider the bifurcation parameters *a* as mediating intrinsic (or within node) dynamics, while the extrinsic (between-node) connectivity is parameterized by the global coupling *G*. In what follows, we optimized the local (bifurcation or intrinsic) parameters to ensure the relative power around each intrinsic frequency band matched the relative power observed in empirical data. This was repeated for several levels (60) of the global (extrinsic) coupling *G*. Having fit the parameters to empirical data, we then inferred the most likely global coupling by seeing how well it predicted a variety of functional integration measures (see below) based upon the empirical data. We then examined the intrinsic bifurcation parameters and tested for differences in their distribution between the four conditions (ON, OFF and both Healthy). We will first describe the three metrics used to find the global coupling that best explained empirical dynamics. We then describe how their distributions were compared over conditions.

#### Agreement between empirical and simulated data

In order to find the best agreement between the empirical and simulated FC’s, three different metrics that capture the static as well as the dynamic organization of brain oscillations were computed across *G* (from 0 to 6 in steps of 0.1) for the mean ON, OFF and both Healthy FC’s. The first one is the static *fitting* between the empirical and simulated FC matrices^73^ computed as the Pearson correlation coefficient of the FC values. The second metric that we used is the *Kolmogorov-Smirnov distance* (ks-d) between the empirical and simulated distributions of phase consistency matrices (see previous paragraphs) reflecting fitting of dynamic aspects of the network. The third and final one is the *metastability* ^71^, which reflects the overall variability of a system’s oscillations across time here derived from the standard deviation of the Kuramoto order parameter^74^:

2 $$R(t)=|\sum _{k=1}^{n}{e}^{i{\phi }_{k}(t)}|/n$$

where φ_*k*_(*t*) represents the phase of all BOLD signals in a given node *k* and *n* is the number of nodes in the network. When *R* = 1 all phases are fully synchronized whereas *R* = 0 means that all phases are complete desynchronized. To do this, it is required to filter the BOLD signals with a band-pass of 0.04–0.07 Hz^75–78^ and further compute the instantaneous phase of each narrowband signal *k* by applying a Hilbert transform in which the phase is analytically represented in:

3 $$s(t)=A(t)\cos (\phi (t))$$

where *s*(*t*) is the analytic representation of a narrowband signal with an instantaneous phase *φ*(*t*)and amplitude *A*(*t*). These two elements are represented as the argument and modulus respectively in a complex signal $z(t)=s(t)+i.H[s(t)]$, where *i* is the imaginary part and H[*s*(*t*)] is the Hilbert transform of *s*(*t*).

#### Comparison of parameter distribution over conditions

For each group, we collected the optimized parameters over 20 coupling values for a total of 900 (20 × 45) optimized parameters. Because we want to investigate what is the impact of DBS on global instead of local bifurcation dynamics and if this reflects a shift towards the bifurcation (~0), we explored and compared the shapes of the parameter distributions in each condition by measuring the kurtosis *k*, which describes the shape of the distribution, the second order raw moment μ_2_, which captures data dispersion from zero and the proportion of parameter values around the bifurcation at a given threshold *thr*.

We then applied a permutation test for *k*, μ_2_ and *thr* to statistically assess recovery and a possible shift to the Healthy regime. For this, we created five joint distributions by pooling together two distributions at a time. These are ON|OFF, ON|H-AM, ON|H-NAM, OFF|H-AM and OFF|H-NAM. The observed statistics (*k*, μ_2_ and *thr*) were compared to those of 10,000 randomized surrogate samples with equal dimension extracted from each of the joint distributions. The proportion of randomized *k*, μ_2_ and *thr* bigger or larger than the observed statistics was used as a significance value.

Next, we computed the ks-d between conditions to estimate the distance between distributions. Also, for each node in the ON and OFF conditions, we computed the optimal mean bifurcation parameter value (±standard deviation) to understand local differences between ON and OFF.

#### Recreating in silico local stimulation

To find which regions contribute more in shifting whole-brain dynamics to that of the Healthy regimes, instead of estimating all local bifurcation parameter values (see modeling paragraphs), we fixed the *a* parameter to a positive value of 0.2 (stable oscillatory regime), one node at a time in DBS OFF and repeated the modeling procedure 1000 times creating evoked bifurcation patterns from DBS OFF for each node. This method allowed, under the context of the current model, recreating stable local oscillatory conditions representative of an *in silico* DBS (Fig. S2). Finally, we estimated the Euclidean distance of the evoked *a* parameter vector (one value per node) from DBS OFF to that of both Healthy regimes given by:

4 $$D=\,\sqrt{\sum _{i=1}^{n}{(aH{e}_{i}-aE{v}_{i})}^{2}}\,$$

The distance *D* portrays which *in silico* stimulation site in DBS OFF brings large-scale dynamics closer to those observed in the Healthy brain. Here, *aHe* represents the Healthy bifurcation reference vector computed as the mean bifurcation parameter per node from either of the two Healthy conditions while *aEv* the evoked DBS OFF vector and *n* is the number of nodes (45 in this case). The node with a fixed parameter value was not included for all Euclidean distance estimations.

## Electronic supplementary material

Supplementary Information
