# Supplementary material for: Uncovering the underlying mechanisms and whole-brain dynamics of deep brain stimulation for Parkinson’s disease
Source: Sci Rep. 2017 Aug 29;7:9882. doi: 10.1038/s41598-017-10003-y (PMC5574998; doi:10.1038/s41598-017-10003-y)
Supplement: Supplementary file 1 — Supplementary Information [file 41598_2017_10003_MOESM1_ESM.pdf]

Uncovering the underlying mechanisms and whole-brain dynamics of deep brain stimulation for Parkinson's disease

Victor M. Saenger[1], Joshua Kahan[2], Tom Foltynie[2], Karl Friston[3], Tipu Z. Aziz[4,5], Alexander L. Green[4,5], Tim J. van Hartevelt[6,7], Joana Cabral[1,6], Angus B. A. Stevner[6,7], Henrique M. Fernandes[6,7], Laura Mancini[8], John Thornton[8], Tarek Yousry[8], Patricia Limousin[2], Ludvic Zrinzo[2], Marwan Hariz[2], Paulo Marques[9,10,11], Nuno Sousa[9,10,11], Morten L. Kringelbach[6,7]* & Gustavo Deco[1,12]

*Corresponding author:
morten.kringelbach@queens.ox.ac.uk

Affiliations

1. Center for Brain and Cognition, Computational Neuroscience Group, Department of Information and Communication Technologies, Universitat Pompeu Fabra, Barcelona, 08018, Spain.
2. Sobell Department of Motor Neuroscience & Movement Disorders, UCL Institute of Neurology, London, WC1N 3BG, United Kingdom.
3. Wellcome Trust Centre for Neuroimaging, Institute of Neurology, University College London, London, WC1N 3BG, United Kingdom.
4. Nuffield Department of Clinical Neurosciences, University of Oxford, Oxford, OX3 9DU, United Kingdom.
5. Nuffield Department of Surgical Sciences, University of Oxford, Oxford, OX3 9DU, United Kingdom.
6. Department of Psychiatry, University of Oxford, Oxford, OX3 7JX, United Kingdom.
7. Center for Music in the Brain, Aarhus University, Aarhus, 8000 Aarhus C, Denmark.
8. Lysholm Department of Neuroradiology, National Hospital for Neurology and Neurosurgery, UCLH NHS Foundation Trust, London, WC1N 3BG, United Kingdom.
9. Life and Health Sciences Research Institute (ICVS), School of Health Sciences, University of Minho, 4710-057 Braga, Portugal
10. ICVS/3B's - PT Government Associate Laboratory, 4710-057 Braga, Portugal
11. Clinical Academic Center, 4710-057 Braga, Portugal
12. Instituci Catalana de la Recerca i Estudis Avanats (ICREA), Universitat Pompeu Fabra, Barcelona, 08010, Spain.

Supplementary Information

Functional connectivity methods

The functional connectivity (FC) analysis of the resting state data from all participants (10 patients, 49 age-matched healthy and 16 non age-matched participants) followed the same analysis pipeline involving extracting functional connectivity time series from the automated anatomical labeling (AAL) parcellation in the native EPI space of each participant. First, we used the AAL template to parcellate the entire brain into 90 regions (cortical and subcortical regions but without the cerebellum)[1]. The linear registration tool from the FSL toolbox (www.fmrib.ox.ac.uk/fsl, FMRIB, Oxford) [2] was used to co-register the EPI image to the T1-weighted structural image. The T1-weighted image was co-registered to the T1 template of ICBM152 in MNI space [3]. The resulting transformations were concatenated and inversed and further applied to warp the AAL template [1] from MNI space to the EPI native space, where interpolation using nearest-neighbor method ensured that the discrete labeling values were preserved. Thus, brain parcellation was conducted in each individual's native space.

We then preprocessed the functional fMRI data using MELODIC (Multivariate Exploratory Linear Decomposition into Independent Components) Version 3.14 [4], part of FSL (FMRIB's Software Library, www.fmrib.ox.ac.uk/fsl). We used the default parameters of this imaging pre-processing pipeline on all participants: motion correction using MCFLIRT [2]; non-brain removal using BET [5]; spatial smoothing using a Gaussian kernel of FWHM 5mm; grand-mean intensity normalization of the entire 4D dataset by a single multiplicative factor and linear de-trending over 50 second intervals. Importantly, MELODIC was used as a preprocessing pipeline only and not to identify and discard components.

We also used tools from FSL to extract and average the time courses from all voxels within each AAL cluster, leaving 90 time series for each AAL region. The grand average of the functional connectivity matrix was constructed using Matlab (The MathWorks Inc.) to compute the pairwise Pearson correlation between all 90 regions, applying Fisher's transform to the r-values to get the z-values for the final 90x90 functional connectivity matrix.

Experimental design

Given the signal artefacts arising from the connection between the electrode lead and the extension cable in the left hemisphere of patients, our analysis was carried out only on data arising from the right hemisphere in both patients and healthy participants to avoid any potential signal artefacts. Hence, from a total of 90 AAL regions, only 45 right hemispheric AAL regions were considered during our analysis. Additionally, empirical resting state fMRI scans as well as simulations of the hemodynamic response while turning DBS on in

patients were named the ON condition while with no stimulation were called the OFF condition. The set of 49 healthy age-matched controls are named the H-AM group. The set of 16 healthy non age-matched controls are named the H-NAM control group.

Whole-Brain Model

The dynamic whole-brain model is reconstructed from the 45 (right hemisphere only) brain regions or nodes (Table 1) based on the structural DTI backbone published by van Hertevelt and colleagues[6]. The underlying structural connectivity matrix C_{ij}, representing the fiber density crossing from area *i* to *j*, is used as a primer for simulating global coupled dynamic interactions between nodes [7]. Here the structural interaction values are scaled so the maximum is 0.2. Local dynamic processes in each node are represented as the normal form of a supercritical Hopf bifurcation (with bifurcation parameter *a*) describing the switching from asynchronous or noisy to regular oscillatory behavior [8]. In a complex plane, the dynamics of a given node *j* are represented by,

$$\frac{dz_j}{dt} = \left[a_j + i\omega_j - \left|z_j^2\right|\right] + \beta\eta_j(t) \qquad (1)$$

in which the argument $z_j = \rho_j e^{i\theta_j} = x_j + iy_j$ and $\eta_j(t)$ is a term to add Gaussian noise with a fixed standard deviation of $\beta = 0.02$ and as previously mentioned, there is a supercritical bifurcation for $a = 0$ and for $a < 0$ the local dynamics have an asynchronous behavior while for $a > 0$ there is a stable oscillation with a frequency $f_j = \omega_j/2\pi$. Further the whole brain dynamics are described by:

$$\frac{dx_j}{dt} = \left[a_j - x_j^2 - y_j^2\right]x_j - \omega_j y_j + G\sum_i C_{ij}\left(x_i - x_j\right) + \beta\eta_j(t) \qquad (2)$$

$$\frac{dy_j}{dt} = \left[a_j - x_j^2 - y_j^2\right]y_j - \omega_j x_j + G\sum_i C_{ij}\left(y_i - y_j\right) + \beta\eta_j(t) \qquad (3)$$

where *G* is a global coupling strength parameter and x_j represents the variable of the modeled BOLD signal in each node *j*. The empirical signals were band-pass filtered with a narrowband of 0.04–0.07 Hz, which contains more functionally relevant information than other bands [9-12]. The intrinsic frequency defined by ω_j in each node was set to the empirical value (for each node) in the range of 0.04 to 0.07 Hz. In other words, we defined our model in terms of the (region specific) intrinsic frequencies observed empirically.

Optimization of the Local bifurcation parameter

To optimize each bifurcation parameter *a,* described previously in the model, the proportion of power in the 0.04-0.07 Hz domain is fitted to the 0.04-0.25 Hz band defined by:

$$p_j = \frac{\int_{0.04}^{0.07} P_j(f)\mathrm{d}f}{\int_{0.04}^{0.25} P_j(f)\mathrm{d}f} \qquad (4)$$

and further updating them by applying a Robbins-Munroe algorithm:

$$a_j = a_j + \eta(\mathrm{P}_j^{emp} - \mathrm{P}_j^{sim}) \qquad (5)$$

until values converge and are stabilized. Here we used fixed value of 0.1 for η.

The impact of UPDRS ratings on brain dynamics

To make sure that the different UPDRS improvement ratings across subjects (see Table 1) had little to no impact on the metrics we used (Figure 1), we calculated the absolute difference between UPDRS-on and off, which represented a clinical improvement magnitude (C) from baseline for each subject:

$$C = |UPDRS_{\ on} - UPDRS_{off}| \qquad (6)$$

We did the same for the integration, mean phase and phase dispersion:

$$I_c = |I_{\ on} - I_{off}| \qquad (7)$$

$$mean\ \varphi_c = |mean\ \varphi_{\ on} - mean\ \varphi_{off}| \qquad (8)$$

$$std\ \varphi_c = |std\ \varphi_{\ on} - std\ \varphi_{off}| \qquad (9)$$

Representing a functional improvement for each metric. We then calculated the correlation between C and I_c, $mean\ \varphi_c$ and $std\ \varphi_c$ to test if C (clinical improvement) added any important confound to our analysis. None of the correlations passed the significance test (p = 0.07,0.15 & 0.21 respectively), suggesting that UPDRS change rate did not affect our results in any significant way and that the improvements seen in DBS ON were not caused or confounded by a tremor effect.

Supplementary References

1 Tzourio-Mazoyer, N. *et al.* Automated anatomical labeling of activations in SPM using a macroscopic anatomical parcellation of the MNI MRI single-subject brain. *NeuroImage* **15**, 273-289, (2002).

2 Jenkinson, M., Bannister, P., Brady, M. & Smith, S. Improved optimization for the robust and accurate linear registration and motion correction of brain images. *NeuroImage* **17**, 825-841, (2002).

3 Collins, D., Neelin, P., Peters, T. & Evans, A. C. Automatic 3D intersubject registration of MR volumetric data in standardized Talairach space. *J. Comp. Ass. Tomogr.* **18**, 192-205 (1994).

4 Beckmann, C. F. & Smith, S. M. Probabilistic independent component analysis for functional magnetic resonance imaging. *IEEE transactions on medical imaging* **23**, 137-152, (2004).

5 Smith, S. M. Fast robust automated brain extraction. *Human Brain Mapping* **17**, 143-155 (2002).

6 van Hartevelt, T. *et al.* Neural plasticity in human brain connectivity: The effects of long term deep brain stimulation of the subthalamic nucleus in Parkinson's disease. *PLoS ONE* **9**, e86496 (2014).

7 Deco, G. *et al.* How local excitation-inhibition ratio impacts the whole brain dynamics. *J Neurosci* **34**, 7886-7898 (2014).

8 Deco, G., Kringelbach, M. L., Jirsa, V. & Ritter, P. The dynamics of resting fluctuations in the brain: metastability and its dynamical cortical core. *bioRxiv*, 065284 (2016).

9 Achard, S., Salvador, R., Whitcher, B., Suckling, J. & Bullmore, E. A resilient, low-frequency, small-world human brain functional network with highly connected association cortical hubs. *J Neurosci* **26**, 63-72 (2006).

10 Biswal, B., Zerrin Yetkin, F., Haughton, V. M. & Hyde, J. S. Functional connectivity in the motor cortex of resting human brain using echo - planar mri. *Magnetic resonance in medicine* **34**, 537-541 (1995).

11 Glerean, E., Salmi, J., Lahnakoski, J. M., Jääskeläinen, I. P. & Sams, M. Functional magnetic resonance imaging phase synchronization as a measure of dynamic functional connectivity. *Brain connectivity* **2**, 91-101 (2012).

12 Ponce-Alvarez, A. *et al.* Resting-state temporal synchronization networks emerge from connectivity topology and heterogeneity. *PLoS computational biology* **11**, e1004100, (2015).

Tables and Figure legends

Fig. S1: Individual functional connectivity matrices for all 10 Parkinson patients. First row depicts the OFF condition while the second the ON condition. Only the right hemisphere is shown.

Fig. S2. Toy representation of *in silico* DBS. A Healthy bifurcation vector *aHe* is used as a reference blueprint. Then, taking the DBS OFF vector as the diseased system, stimulation is performed by fixing the parameter *a* for each node to the positive oscillatory regime and simulating the global dynamics given this forced condition. This process is repeated 1000 times to enhance variability. Then, the Euclidean distance is computed between all evoked (stimulated) OFF vectors *aEv* and *aHe.* This distance represents the agreement between the stimulated diseased and the Healthy brain. Lower *D* values reflect stimulation sites pushing the system closer to the Healthy regime.

Fig. S3. Euclidean distance to Healthy (non age-matched) from OFF after artificial DBS. Color map depicted in the left represents the distance to H-NAM from OFF after artificial DBS in each of the 45 nodes across all simulations. The top 5 regions with the lowest mean distance are indicated with a red arrow. The mean Euclidean distance ranked from lowest to highest and the top 5 nodes are depicted in the right plot.

	Tremor		Rigidity		Bradykinesia		Total	
	Off/OFF	Off/ON	Off/OFF	Off/ON	Off/OFF	Off/ON	Off/OFF	Off/ON
1	9	0	11	2	25	14	53	21
2	11	4	4	0	15	6	33	10
3	1	0	8	0	34	15	60	20
4	4	0	12	1	30	14	51	17
5	6	0	12	7	20	12	45	26
6	2	0	9	1	31	14	52	19
7	8	2	9	3	23	13	51	23
8	1	0	5	0	30	18	46	25
9	4	3	11	12	20	18	44	42
10	1	4	15	12	25	18	53	44

Supplementary Table 1: Detailed breakdown of clinical symptoms. Scores from UPDRS part III subsections that were directly attributed to tremor, rigidity or bradykinesia were grouped to demonstrate that most patients showed improvements in tremor, rigidity and bradykinesia when stimulation was turned ON. Scores were collected in the "off" medication state. Total refers to the sum of the bradykinesia, rigidity and tremor subdomains, as well as postural and other components of the UPDRS-III.

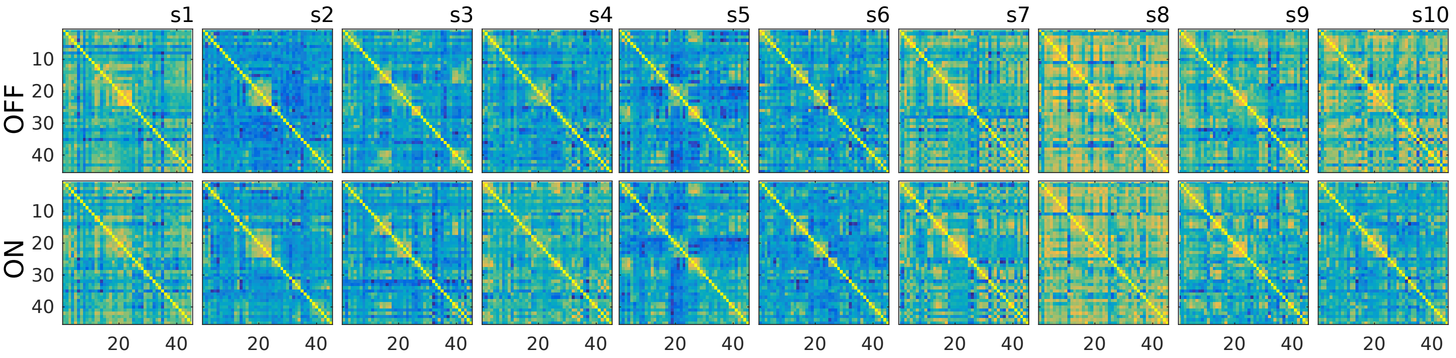

Fig. S1. Individual functional connectivity matrices for all 10 Parkinson patients.

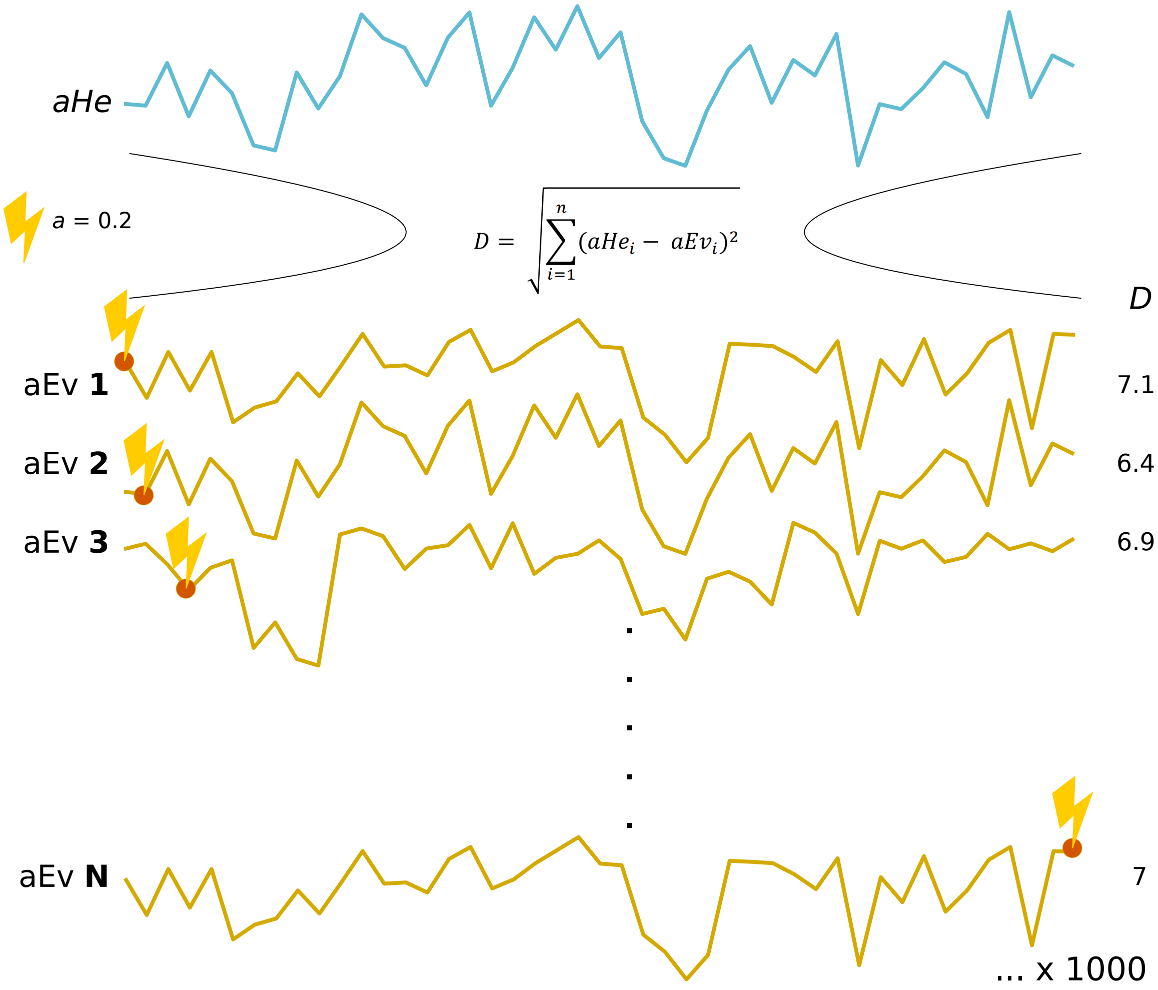

Fig. S2. Toy representation of *in silico* DBS. A Healthy bifurcation vector *aHe* is used as a reference blueprint.

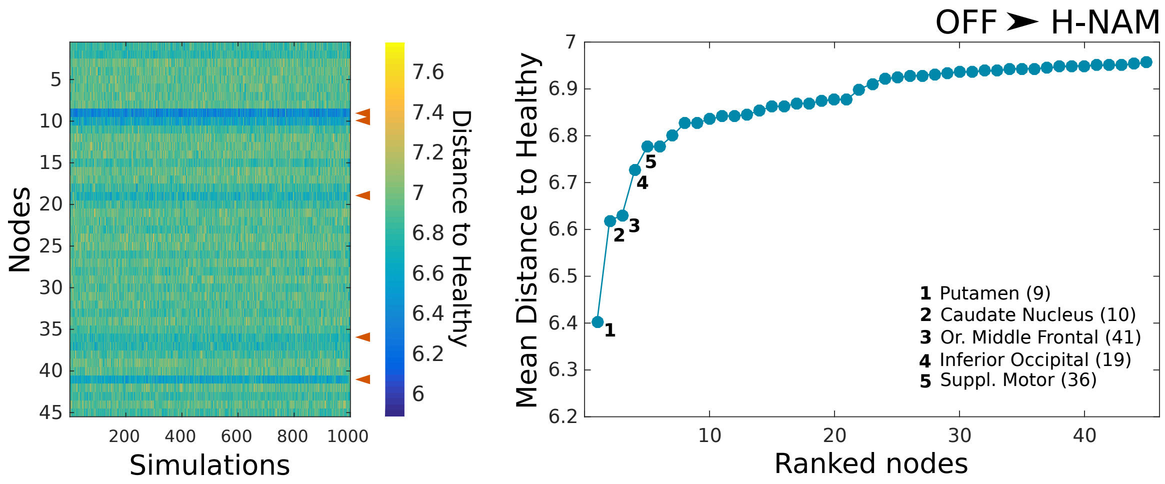

Fig. S3. Euclidean distance to Healthy (non age-matched) from OFF after artificial DBS
